# Cefdinir binding to a class A β-lactamase revealed by serial cryo-crystallography

**DOI:** 10.1107/S2059798326003992

**Published:** 2026-05-20

**Authors:** Gargi Gore, Andreas Prester, Kim Bartels, David von Stetten, Eike C. Schulz

**Affiliations:** a University Medical Centre Hamburg-Eppendorf (UKE) Hamburg Germany; b Institute for Nanostructure and Solid State Physics, University of Hamburg, Hamburg, Germany; c European Molecular Biology Laboratory (EMBL), Hamburg, Germany; d Max-Planck-Institute for the Structure and Dynamics of Matter Hamburg Germany; National Hellenic Research Foundation, Greece

**Keywords:** serial crystallography, CTX-M-14, cefdinir, cephalosporins, antibiotic resistance

## Abstract

One of the most common resistance mechanisms against antibiotics employed by Gram-negative bacteria involves the production of β-lactamases, resulting in rapid hydrolysis of the antibiotic. Extensive use of the early-generation cephalosporins led to the rise of extended-spectrum β-lactamases such as CTX-Ms. Cefdinir is an extended-spectrum third-generation cephalosporin administered since the late 1990s; despite this, there is no reported 3D structure of the antibiotic bound to any β-lactamase or penicillin-binding protein in the PDB. Here, we report the X-ray crystallographic structure of cefdinir-bound CTX-M-14 E166A mutant obtained via serial cryo-crystallography.

## Abstract 2

Serial cryo-crystallography reveals the structure of the extended-spectrum β-lactamase CTX-M-14 in complex with the third-generation cephalosporin antibiotic cefdinir.

## 1. Introduction

Widespread antibiotic resistance is turning into a public health issue, claiming millions of lives annually (Ikuta *et al.*, 2022 ▸; Murray *et al.*, 2022 ▸). An alarming study showed that in 2019 alone, almost two million deaths could be attributed to multidrug-resistant bacteria (Murray *et al.*, 2022 ▸). Various pathogenic bacteria can employ a repertoire of different resistance mechanisms, including, but not limited to, active expulsion of the antibiotic from the cell, generation of alternative metabolic pathways, modification of the target site and enzymatic alteration of the antibiotic (Urban-Chmiel *et al.*, 2022 ▸).

One of the most important and prevalent resistance mechanisms exhibited by Gram-negative infectious bacteria is the production of β-lactamases, which hydrolyse β-lactam rings and thereby degrade antibiotics (Tooke *et al.*, 2019 ▸; Wilke *et al.*, 2005 ▸; Cantón *et al.*, 2012 ▸). β-Lactam antibiotics disrupt peptidoglycan cross-linking during cell-wall synthesis, making the cell susceptible to lysis and death. Extensive and improper use of β-lactam antibiotics in human health and agriculture has increased the selective pressure, leading to a growing number of resistant bacterial strains (Bradford, 2001 ▸; Bonnet, 2004 ▸). Successful proliferation of bacteria with a wide multidrug-resistance profile has been further supported by horizontal gene transfer (HGT), wherein mobile genetic elements such as plasmids and transposons harbouring antibiotic-resistance genes are exchanged between bacteria (Van Hoek *et al.*, 2011 ▸). Thus, both the natural selection of mutants with high substrate specificity and the transfer of resistance genes from the surrounding metagenome led to the evolution of β-lactamases with an extended-substrate spectrum (ESBLs) in the Enterobacteriaceae family (D’Andrea *et al.*, 2013 ▸). ESBLs efficiently hydrolyse penicillins, as well as broad-spectrum cephalosporins (such as cefotaxime, ceftriaxone and ceftazidime) and monobactams (for example aztreonam), but do not effectively hydrolyse cephamycins or carbapenems and can be inhibited by some inhibitors (Cantón *et al.*, 2012 ▸; van Hoek *et al.*, 2011 ▸).

**Figure 1:**
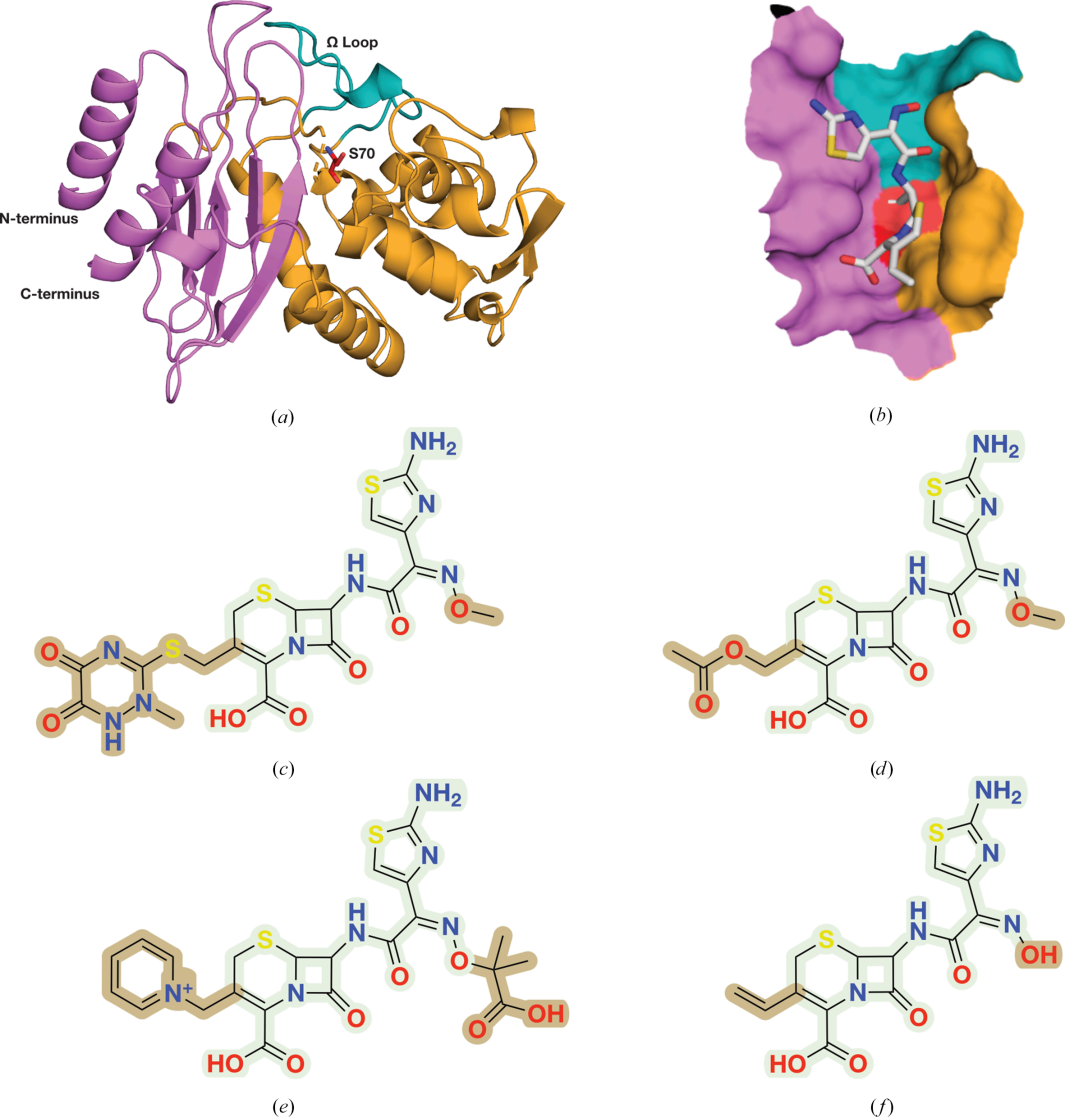
Model system, target ligand and structural homology between selective cephalosporins. (*a*) CTX-M-14 β-lactamase. The catalytically important active-site residue Ser70 and the functionally relevant Ω loop (residues 160–180) are highlighted. (*b*) Cefdinir in the active site of CTX-M-14 E166A. (*c*) Ceftriaxone. (*d*) Cefotaxime. (*e*) Ceftazidime. (*f*) Cefdinir. Differences in the side chain are highlighted.

In Enterobacteriaceae, the CTX-M β-lactamases have largely surpassed other Ambler class A ESBL enzymes in terms of ubiquity (Cantón *et al.*, 2012 ▸). The CTX-M family consists of five main clusters, CTX-M-1, CTX-M-2, CTX-M-8, CTX-M-9 and CTX-M-25, which differ from each other in amino-acid sequence by ≥10% (Cantón *et al.*, 2012 ▸). Out of the five, CTX-M-1 and CTX-M-9 are the most widespread clusters. The focus of the present study is CTX-M-14 (Fig. 1 ▸*a*), which belongs to the CTX-M-9 subgroup and is one of the most important and hence extensively studied enzymes of the CTX-M family (D’Andrea *et al.*, 2013 ▸). CTX-M-14 β-lactamases can effectively hydrolyse most cephalosporins and penicillins; they also hydrolyse carbapenems, albeit with a reduced efficiency (Ishii *et al.*, 2007 ▸). Essentially, class A enzymes hydrolyse the peptide-mimicking bond in the β-lactam ring of the antibiotic by stepwise acylation and deacylation. During the first part of the reaction, the active-site residue Ser70 attacks the carbonyl carbon of the β-lactam, cleaving the amide bond, which protonates the β-lactam nitrogen, forming an acyl-enzyme intermediate. In the second part of the reaction, Glu166 then activates a catalytic water, which attacks the carbonyl carbon of the ester bond between the oxygen of Ser70 and the β-lactam ring. In turn, this results in hydrolysis and release of the inactivated antibiotic and regeneration of the catalytically competent enzyme (Ambler, 1980 ▸; Drawz & Bonomo, 2010 ▸; Hata *et al.*, 2006 ▸). Consequently, modification of either Ser70 or Glu166 impairs the catalysis and aids in trapping intermediates along the reaction-coordinate pathway.

Third-generation cephalosporins are the most commonly prescribed cephalosporins against infections caused by both Gram-positive and Gram-negative bacteria. These antibiotics consist of a six-membered dihydrothiazine ring fused to the β-lactam ring. The C3, C4 and C7 functional groups determine their antimicrobial activity and chemical properties (El-Shaboury *et al.*, 2007 ▸). Cefdinir is a semi-synthetic, extended-spectrum oximino-cephalosporin which has been prescribed against bacterial pneumonia, other respiratory-tract infections, otitis media and skin infections since the late 1990s (Guay, 2002 ▸). Although cefdinir is more susceptible to hydrolysis by β-lactamases than larger cephalosporins such as ceftazidime and ceftriaxone, it remains clinically relevant due to its oral administrability.

Originally identified as cefotaximases, CTX-Ms readily hydrolyse smaller oximino-cephalosporin derivatives, but perform poorly against bulkier varieties such as ceftazidime and ceftriaxone. However, recent studies reveal that naturally occurring single-amino-acid substitutions in CTX-Ms, such as A77V, N106S, P167S/T/Q and D240G, result in a drastic increase in the cephalosporin-hydrolysing ability (Bonnet, 2004 ▸; Chen *et al.*, 2005 ▸; Novais *et al.*, 2008 ▸; Both *et al.*, 2017 ▸; Patel *et al.*, 2018 ▸). It is therefore crucial to determine the exact nature of the interaction between enzyme and substrate with clinically relevant cephalosporins, which could be helpful for future structure-based drug development.

Previously determined complexes of cefdinir homologues such as ceftazidime and ceftriaxone have provided invaluable insight into ESBL active-site plasticity (Brown *et al.*, 2020 ▸; Patel *et al.*, 2017 ▸, 2018 ▸; Lu *et al.*, 2023 ▸). Recent studies have also explored the idea of a cefdinir–inhibitor combination for improved bactericidal efficiency against β-lactamases (Srivastava *et al.*, 2021 ▸). However, there is currently no reported crystallographic analysis of any β-lactamase in complex with the clinically relevant cephalosporin cefdinir. To address this gap, we utilized serial cryo-crystallography and determined the structures of wild-type apo CTX-M-14, apo CTX-M-14 E166A and cefdinir bound to CTX-M-14 E166A.

## 2. Materials and methods

### 2.1. Protein purification and crystallization

The CTX-M-14 WT and CTXM-14 E166A genes were both synthesized and cloned into a pET-24a(+) vector (BioCat GmbH, Heidelberg, Germany) with a kanamycin selection marker. The plasmids were transformed into *Escherichia coli* strain BL21 (DE3) and grown in LB (Luria Miller) medium supplemented with 50 µg ml^−1^ kanamycin at 37°C until an OD_600_ of 0.6–0.8 was reached. Protein expression was induced by the addition of isopropyl β-d-1-thiogalactopyranoside to a final concentration of 175 µ*M*, after which the cells were incubated at 37°C for 4 h. The cells were harvested by centrifugation (5500*g*, 10 min, 4°C) and the pellets were stored at −20°C until purification. For purification, the cell pellet was resuspended in purification buffer (20 m*M* MES pH 6), followed by sonication for cell lysis. Cell debris was separated by centrifugation (20 000*g*, 1 h, 4°C). The cleared supernatant was dialysed overnight against a large volume of purification buffer at 4°C using a 6–8 kDa molecular-weight cutoff membrane. Both proteins were purified using cation-exchange chromatography (5 ml HiTrap SP FF, Cytiva) and eluted using a gradient of 0–50 m*M* NaCl in 20 m*M* MES pH 6 over five column volumes. The proteins were then concentrated to 22 mg ml^−1^ using 10 kDa centrifugal filter units (Amicon Ultra-15).

For crystallization of both the wild type and the activity-impaired mutant E166A, protein solution (22 mg ml^−1^) was mixed with 45%(*v*/*v*) crystallizing agent [40%(*w*/*v*) PEG 8000, 200 m*M* LiSO_4_, 100 m*M* sodium acetate pH 4.5] and with 5%(*v*/*v*) undiluted seed-stock solution to induce micro-crystallization. This resulted in crystals with a homogeneous size distribution of ∼11–15 µm overnight.

For soaking experiments, cefdinir was dissolved in the stabilization buffer [28%(*w*/*v*) PEG 8000, 140 m*M* LiSO_4_, 70 m*M* sodium acetate, 6 m*M* MES pH 4.5] to reach a concentration of about 50 m*M*. The ligand did not dissolve completely; therefore, the supernatant was mixed with the crystal slurry in a 1:3 ratio with 12%(*v*/*v*) 2,3-butanediol as a cryoprotectant.

### 2.2. Structure determination

About 2 µl of microcrystalline sample was pipetted onto a SPINE-standard mesh loop with a 400 µm mesh area having 10 µm openings (MiTeGen, MicroMesh). After deposition, the mesh was blotted from behind using Whatman paper to remove excess mother liquor. The initially established cryogenic (at 77 K) serial data collection involved raster scanning with rotational exposure (Gati *et al.*, 2014 ▸). However, we collected still diffraction data using a previously described workflow (Mehrabi *et al.*, 2023 ▸) available in *MXCube* (Oscarsson *et al.*, 2019 ▸) at the P14 beamline of PETRA-III (EMBL Hamburg Unit, Germany).

In brief, a micro-focused 7 × 3 µm (FWHM) sized X-ray beam with an energy of 12.7 keV (0.9763 Å), a flux of 2 × 10^13^ photon s^−1^ and an exposure time of 7.5 ms per image was used during data collection with an EIGER2 CdTe 16M detector (Dectris, Baden-Daettwil, Switzerland). During data collection, a grid with spacing matching the dimensions of the beam was drawn over the whole micro-mesh sample, giving rise to several thousand still diffraction images, which were processed using *CrystFEL* with the *XGANDALF* indexing routine (Gevorkov *et al.*, 2019 ▸; White *et al.*, 2012 ▸). Structures were solved by molecular replacement in *Phaser* using PDB entry 6gth (Wiedorn *et al.*, 2018 ▸) as a search model for CTX-M-14 (McCoy *et al.*, 2007 ▸). Structure refinement was performed with iterative cycles of *phenix.refine* and manual model building in *Coot* v.0.9 (Adams *et al.*, 2004 ▸; Emsley & Cowtan, 2004 ▸; Emsley *et al.*, 2010 ▸). Figures were generated with *PyMOL* (Schrödinger) and *ChemDraw* (Revvity Signals Software, Waltham, USA). Table 1 ▸ summarizes the data-collection and refinement statistics.

## 3. Results and discussion

### 3.1. Cryo-SSX

Serial synchrotron crystallography under cryo-conditions (cryo-SSX) is a useful alternative to conventional single-crystal cryo-crystallography when large crystals are difficult to produce but microcrystals are available. When solubility issues with the ligand result in problematic soaking times for larger crystals, microcrystals can be a viable alternative, thanks to their favourable surface-to-volume ratio. Another significant advantage is the reduction in radiation damage that can be achieved through serial data collection, whereby the dose is distributed across thousands of crystals. Unlike SSX at room temperature, cryo-SSX offers the advantage of standardized sample handling: samples can be prepared before the beamtime, loaded onto commercially available holders and shipped. This standardization enables access to high-throughput data-collection workflows, such as those found at many synchrotron beamlines.

On the other hand, the cryo-SSX method presents challenges related to the sample: if the crystal density is too high, this can lead to overlapping diffraction patterns. Conversely, if the crystal density is too low, this can result in insufficient orientation diversity. Such a lack of orientational multiplicity among the indexed microcrystals might cause inferior crystallographic data-quality metrics (for example *R* factors) during model refinement than the resolution of the dataset would imply for canonical rotation data. Unfortunately, a clear metric indicating this orientational bias is currently lacking. While further data-analysis tools will be required in the future to ascertain the cause, we have attempted to circumvent these problems by careful sample preparation and sampling a larger number of diffraction patterns than typically required for room-temperature SSX (Moreno-Chicano *et al.*, 2019 ▸; Gorel *et al.*, 2021 ▸; Mehrabi *et al.*, 2021 ▸). We achieved larger sampling by depositing about 2 µl sample per mesh (to avoid dehydration after blotting), with repeats of each dataset.

Here, we have determined the structures of the ESBL CTX-M-14, of its activity-impaired E166A mutant and of CTX-M-14 E166A in complex with the third-generation extended-spectrum cephalosporin cefdinir (Figs. 1 ▸ and 2 ▸) via cryo-SSX. During data collection, we obtained 105 490 diffraction images for the wild-type apo structure, of which 58 030 were indexable. Similarly, out of the 157 342 diffraction images for the E166A apo structure, 81 966 were indexable. We obtained 61 422 diffraction patterns for the cefdinir-bound complex, of which 30 491 could be successfully indexed. For all three structures, we collected and merged two meshes. All structures were solved by molecular replacement with one monomer in the asymmetric unit and were refined to comparable and reasonable data-quality parameters (Table 1 ▸).

### 3.2. Influence of the E166A mutation on the apo structure of CTX-M-14

The activity-impaired CTX-M-14 mutant E166A crystallized with one monomer per asymmetric unit. The protein adopts the canonical, previously observed two-domain structure (Lee *et al.*, 2025 ▸). Superposition with the wild-type protein demonstrates a root-mean-square deviation (r.m.s.d.) of 0.15 Å, thereby substantiating the observation of overall high structural agreement between the mutant and the wild-type enzyme.

At the site of the mutation, the backbone structures can be superimposed with minimal differences. One significant difference between the WT and E166A mutant is the position of the catalytic water molecule essential for the successful completion of ligand hydrolysis. In the case of the E166A mutant, the catalytic water, which is present near the active Ser70 in the WT, is displaced ∼1.5 Å away due to a lack of coordination with Glu166. This in turn prevents the deacylation step and traps the acyl-enzyme intermediate. The E166A mutation seems to induce a slight backbone shift in residues Thr165–Leu169, especially in Pro167 (0.4 Å). Nevertheless, this shift does not prevent cefdinir from being placed in its current position, supporting the hypothesis that the reduction in catalytic activity can be assigned to functional rather than structural differences between the proteins.

### 3.3. CTX-M-14 E166A–cefdinir interaction

After molecular replacement of the CTX-M-14 E166A–cefdinir complex, we determined strong difference electron density in the active site. The electron density encompasses the entire ligand and is clearly connected to the active-site Ser70, indicating a hydrolysed, covalently bound cefdinir resembling the acyl-enzyme intermediate.

**Figure 3:**
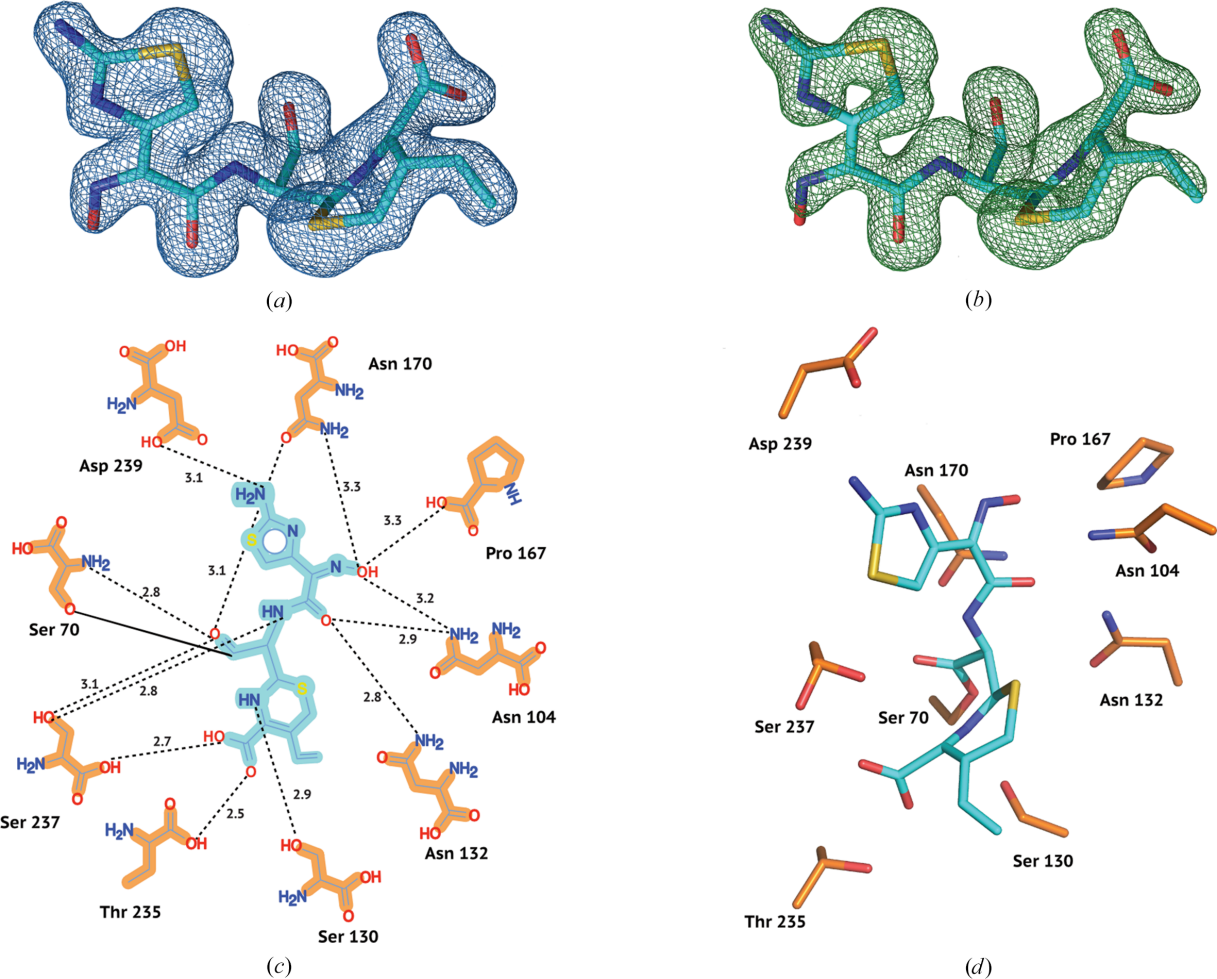
Electron density and conformation of cefdinir at cryo temperature. (*a*) 2*F*_o_ −*F*_c_ map shown at an r.m.s.d. of 1.0. (*b*) Polder omit map shown at an r.m.s.d. of 3.0. (*c*) 2D projection of cefdinir and the contact residues showing interatomic distances. Hydrogen bonds are represented as black dotted lines with their distances given in Å. (*d*) Cefdinir conformation in the CTX-M-14 active site.

Cefdinir, as is characteristic of cephalosporins, contains a core in which a four-membered β-lactam ring is fused to a six-membered dihydrothiazine ring. The C3 position of the core is extended via a vinyl group, while the C4 carbon of the core is attached to a carboxyl group. Additionally, the C7 carbon of the core is bound to an aminothiazolyl-oxime-acetamido side chain (Fig. 1 ▸*f*). Similar to the interactions observed in previously determined CTX-M-14–substrate complexes, cefdinir also interacts with the typical active-site residues of CTX-M-14. Canonically, ligands bound to CTX-M-14 active sites are stabilized by a hydrogen-bond network with the surrounding Ser70, Asn104, Ser130, Asn132, Pro167, Asn170, Thr235, Ser237 and Asp239 residues, which is also reflected in the cefdinir complex (Fig. 3 ▸). Usually, hydrogen bonds between the carbonyl oxygen of the β-lactam and the main-chain nitrogens of Ser70 and Ser237 stabilize the developing negative charge on the tetrahedral intermediate during acylation (Matagne *et al.*, 1998 ▸). This pattern is also observed in the cefdinir complex, where 2.8 and 2.9 Å hydrogen bonds are maintained between the carbonyl oxygen and the main-chain nitrogens of Ser70 and Ser237, respectively. The Ω loop encloses the active site in class A β-lactamases (Fetrow, 1995 ▸; Ibuka *et al.*, 1999 ▸; Poirel *et al.*, 2001 ▸). Important interactions necessary to preserve the structural integrity of the Ω loop (Ibuka *et al.*, 2003 ▸; Chen *et al.*, 2005 ▸) are also conserved in the cefdinir complex. For instance, salt bridges between Arg164 and Asp179, between Arg161 and Asp163 and between Asp176 and Arg178 are present. Additionally, hydrogen bonds between Arg164 and Thr171 and between Ala166 and Asn136 (in contrast to the hydrogen bond between Glu166 and Asn170 in the wild-type enzyme) are also observed. Thus, the Ω loop is well preserved in the E166A mutant and shows no significant conformational variation upon ligand binding. Additionally, the conformation of loops 103–106 and 213–220 has been reported to be important for the efficient binding and hydrolysis of cephalosporins (Patel *et al.*, 2018 ▸; Lu *et al.*, 2023 ▸). The crystal structure shows that these loops are well preserved in the CTX-M-14 E166A–cefdinir complex.

### 3.4. Comparison to other cephalosporins

Chemically, the differences between broad-spectrum third-generation cephalosporins present in the PDB, such as ceftriaxone, cefotaxime and ceftazidime, and cefdinir are located in the C3 and C7 side chains (highlighted in Figs. 1 ▸*c*–1 ▸*f*). Ceftriaxone has a thiotriazinedione (C3) and an aminothiazole-methoxyimino group (C7) (Fig. 1 ▸*c*), cefotaxime has an acetoxymethyl group (C3) and an aminothiazole-methoxyimino group (C7) (Fig. 1 ▸*d*), ceftazidime has a pyridinium group (C3) and an aminothiazole-methoxyimino group with a carboxypropyl tail (C7) (Fig. 1 ▸*e*) and cefdinir has a vinyl group (C3) and an aminothiazolyl-oxime-acetamido side chain (C7) (Fig. 1 ▸*f*). To understand how cefdinir compares with other cephalosporin derivatives, we compared our structure with those of CTX-M-14 complexes with cefotaxime (Soeung *et al.*, 2020 ▸; PDB entry 7k2w; E166A/K234R mutant) and ceftazidime (Patel *et al.*, 2017 ▸; PDB entry 5u53; E166A mutant). The previously determined ceftriaxone structure is bound to BlaC and thus is excluded from the present comparison.

**Figure 4:**
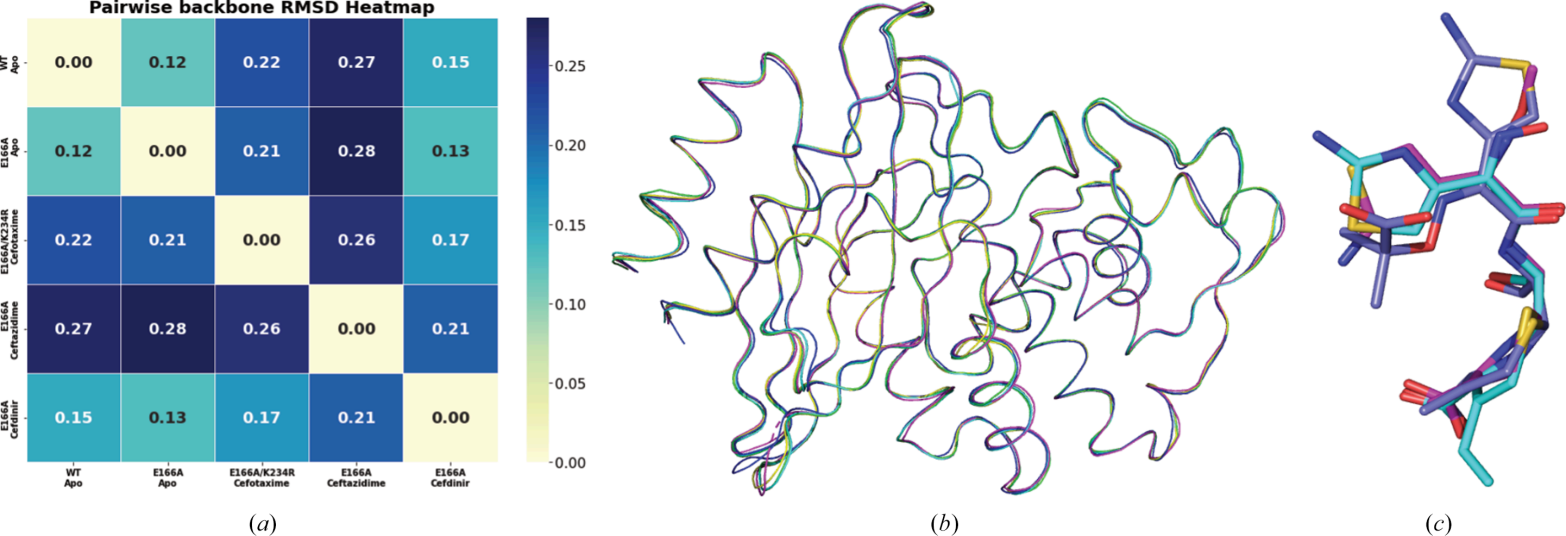
Structural comparison of homologues. (*a*) Pairwise C^α^ categorical heatmap. (*b*) Ribbon representation of superposed wild-type apo (yellow), E166A apo (green), cefotaxime (magenta, PDB entry 7k2w), ceftazidime (blue, PDB entry 5u53) and cefdinir (cyan, PDB entry 9tld) complex structures. (*c*) Superposed cefotaxime (magenta, PDB entry 7k2w), ceftazidime (blue, PDB entry 5u53) and cefdinir (cyan, PDB entry 9tld)

To understand global structural differences between the different ligand complexes, we calculated the C^α^ r.m.s.d. between the apo wild-type, apo E166A, E166A/K234R–cefotaxime, E166A–ceftazidime and E166A–cefdinir structures (Fig. 4 ▸*a*). The categorical heatmap shows C^α^ r.m.s.d. values with a maximum of ∼0.3 Å, which indicates no significant backbone changes between the complexes and is reflected in a good superposition of the structures (Fig. 4 ▸*b*). As the heatmap indicates, the structures overlay quite well. The structurally and functionally important Ω loop does not show pronounced differences when aligned. The only striking difference between the five structures is the loop enclosing residues 252–257, which are not part of the canonical catalytically important residues. Interestingly, our two SSX apo structures and the acyl-enzyme complex align very well in this region, whereas the cefotaxime and ceftazidime datasets differ from each other as well as our structures. Cryo-trapping or differences in crystallization conditions could have introduced this variation.

The CTX-M-14 cefotaxime and ceftazidime complexes also exhibit interactions similar to the cefdinir complex (Fig. 3 ▸*c*). Superposition of the ligands in the active site of CTX-M-14 (Fig. 4 ▸*c*) indicates a partially flipped binding pose of ceftazidime. This different ligand orientation may be caused by the slightly bulkier C7 side chain in ceftazidime. It is worth noting that the C3 side chains of cefotaxime (acetoxymethyl) and ceftazidime (pyridinium) are likely to have been eliminated during the acylation and formation of the acyl-enzyme intermediate, as observed in other serine β-lactamase structures in complex with cephalosporin antibiotics (Hamilton-Miller, 1994 ▸; Patel *et al.*, 2017 ▸; Tooke *et al.*, 2021 ▸). However, there was no elimination in the case of cefdinir, even though an acyl-enzyme intermediate was formed. It is likely that the presence of a vinyl side chain rather than a good leaving group at C3 prevents fragmentation.

### 3.5. Conformational differences induced by cefdinir binding

**Figure 5:**
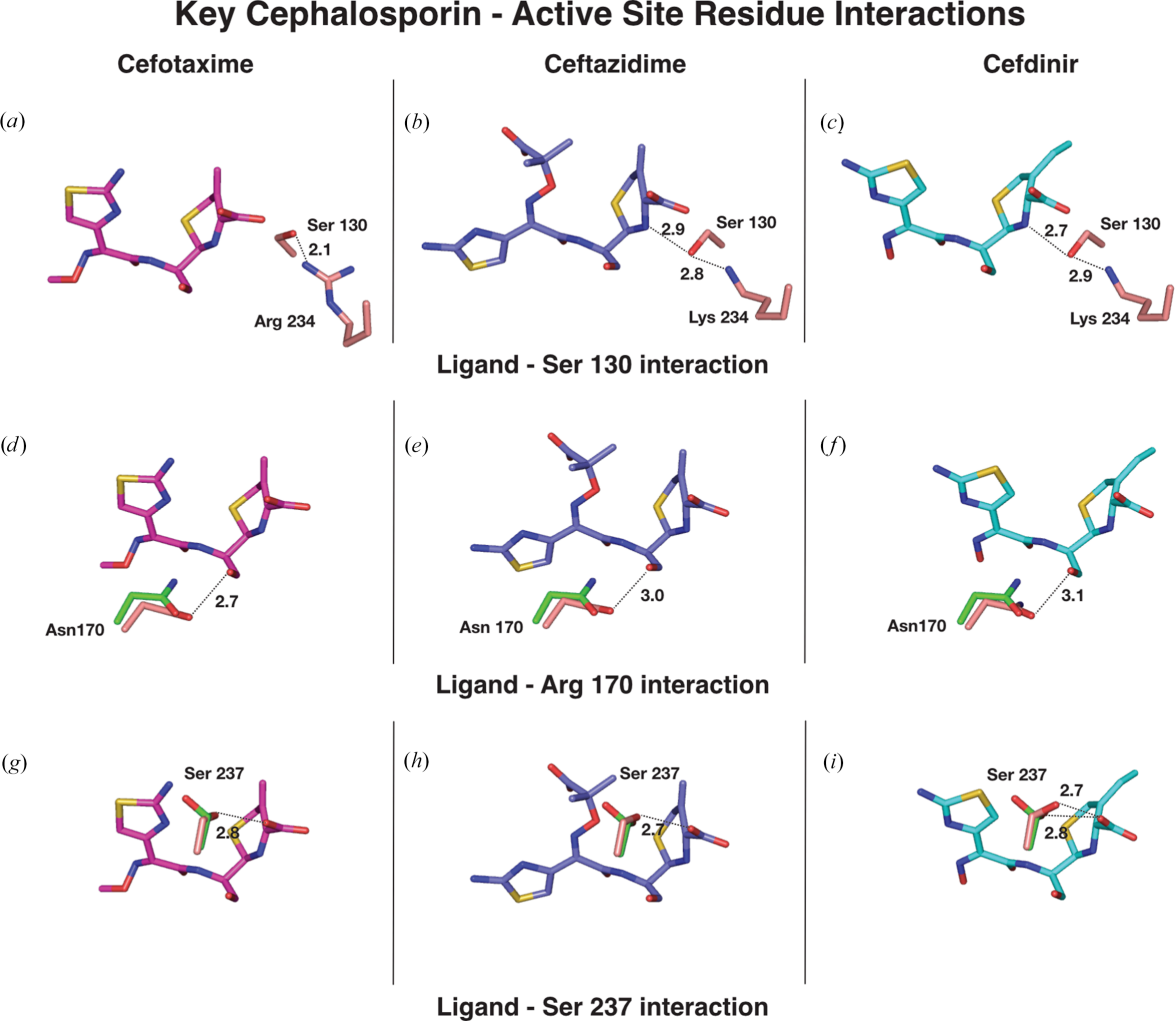
Comparison of key ligand–residue interactions for cefotaxime, ceftazidime and cefdinir. (*a*) Residues Ser130 and Lys234 interacting with cefotaxime. (*b*) Residues Ser130 and Lys234 interacting with ceftazidime. (*c*) Residues Ser130 and Lys234 interacting with cefdinir. (*d*) Residue Asn170 interacting with cefotaxime. (*e*) Residue Asn170 interacting with ceftazidime. (*f*) Residue Asn170 interacting with cefdinir. (*g*) Residue Ser237 interacting with cefotaxime. (*h*) Residue Ser237 interacting with ceftazidime. (*i*) Residue Ser237 interacting with cefdinir.

A difference can be observed between the three (Fig. 5 ▸) acyl-enzyme intermediate complexes (Figs. 5 ▸*a*, 5 ▸*b* and 5 ▸*c*) around Ser130, a residue contributing to both the substrate binding and catalysis of cephalosporins (Lu *et al.*, 2023 ▸). In the case of the E166A–ceftazidime and E166A–cefdinir complexes, Ser130 forms hydrogen bonds with Lys73, Lys234 (which in turn interacts with a water molecule and Thr235) and the dihydrothiazine ring nitrogen. In the E166A/K234R–cefotaxime complex, as a result of the K234R mutation, Ser130 is reoriented by almost 90° to form a hydrogen bond with Arg234, losing the contact with Lys73, but in turn hydrogen-bonds to the carboxylate oxygen of the ligand.

Figs. 5 ▸(*d*), 5 ▸(*e*) and 5 ▸(*f*) show Asn170 from each complex (salmon) superposed with Asn170 from the apo E166A (green) structure. It was hypothesized that in CTX-Ms the hydrogen bond between Ω-loop residue Asn170 (O) and Asp240 (N) breaks to widen the active site to accommodate cephalosporins such as cefotaxime in the binding pocket (Adamski *et al.*, 2015 ▸; Delmas *et al.*, 2010 ▸). Thus, this hydrogen bond remains intact in wild-type apo CTX-M-14 but is disrupted in substrate-bound structures. Similar to the cefotaxime and ceftazidime complexes, this bond is missing in the cefdinir complex, indicating that cefdinir also leads to a widening of the active site. As a consequence of this, the Asn170 side-chain orientation is slightly altered in cephalo­sporin-bound structures compared with the apo WT and CTX-M-14 E166A enzymes. In all cephalosporin-bound structures, Asn170 is positioned to interact with the carboxyl oxygen of the β-lactam.

The conservation of residues Ser237 and Arg274 throughout the CTX-M family and corresponding published studies have revealed that the two residues work in synchrony to stabilize bound cephalosporins (Adamski *et al.*, 2015 ▸; Pérez-Llarena *et al.*, 2008 ▸; Gazouli, Tzelepi, Markogiannakis *et al.*, 1998 ▸; Delmas *et al.*, 2010 ▸; Gazouli, Tzelepi, Sidorenko *et al.*, 1998 ▸). The side chain of Ser237 interacts with the C4 carboxyl of the ligand in all three cases (Figs. 5 ▸*g*, 5 ▸*h* and 5 ▸*i*). The figures also show apo E166A Ser237 (green) superposed on Ser237 of the ligand-bound enzyme (salmon). The apo Ser237 residue assumes two conformations: one is clearly directed about 150° away from the orientation resembling Ser237 of the ligand-bound enzyme. Unlike in the cefotaxime and ceftazidime complexes, in the E166A–cefdinir complex we observe two orientations of the Ser237 side chain. This implies an intrinsic mobility of Ser237, which is potentially important for substrate binding and stabilization.

**Figure 6:**
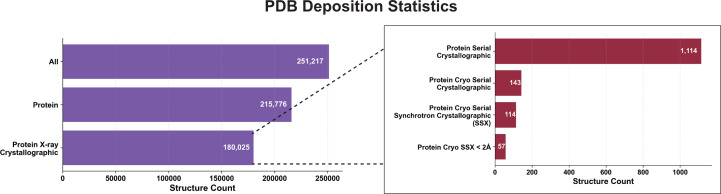
Breakdown of PDB depositions per category. The statistics show the minuscule number of protein cryo-SSX structures in the PDB at the time of writing (Burley *et al.*, 2022 ▸).

To conclude, our study reveals the acyl-enzyme complex between the cephalosporin cefdinir and CTX-M-14 E166A obtained via cryo-SSX. Comparison with structural homologues confirms a comparable binding mode. Moreover, considering that currently only about 0.05% of protein structures in the PDB are obtained via cryo-SSX (Fig. 6 ▸), this study emphasizes that cryo-SSX is a valid alternative to canonical single-crystal data collection and can be exploited for the reliable characterization of protein–ligand interactions.

## Supplementary Material

PDB reference: CTX-M-14, 9tkx

PDB reference: E166A mutant, 9tky

PDB reference: E166A mutant, complex with cefdinir, 9tld

## Figures and Tables

**Figure 2:**
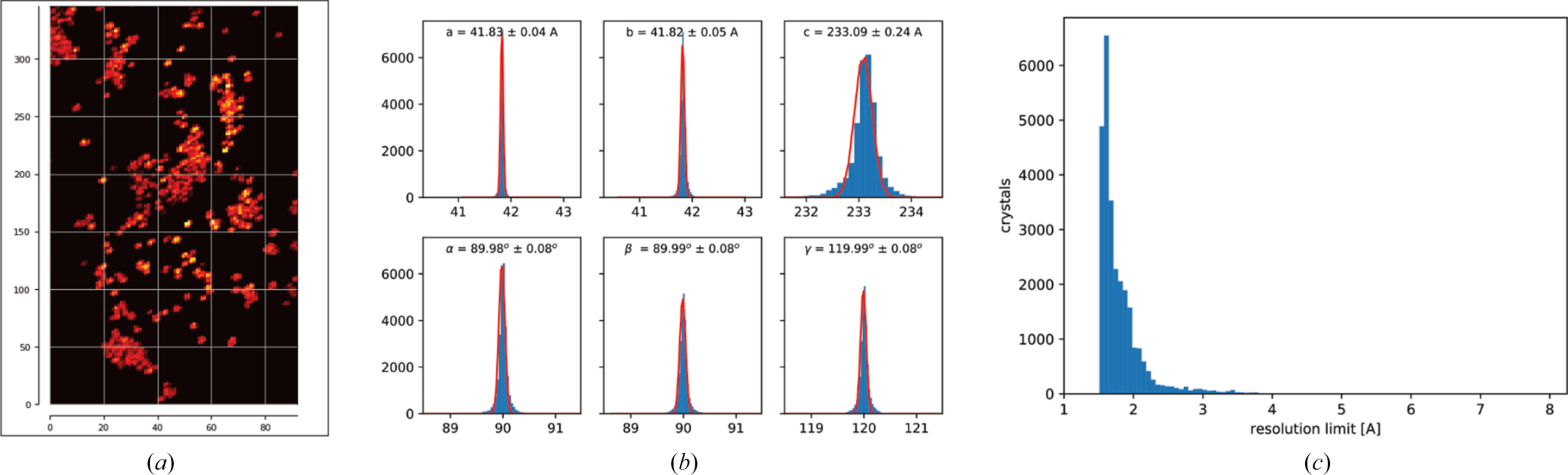
Selective example of typical serial cryo data-collection and processing output. (*a*) The heatmap of crystal hits onto the mesh. (*b*) Unit-cell distribution of the microcrystals. (*c*) Resolution distribution of the identified microcrystals.

**Table 1:** Data-collection and refinement statistics Values in parentheses are for the outer shell.

	Wild-type apo (PDB entry 9tkx)	E166A apo (PDB entry 9tky)	Cefdinir complex (PDB entry 9tld)
Data collection			
Space group	*P*3_2_21	*P*3_2_21	*P*3_2_21
*a*, *b*, *c* (Å)	41.82, 41.81, 232.36	41.78, 41.76, 232.92	41.82, 41.82, 233.09
α, β, γ (°)	89.97, 89.99, 119.98	89.97, 89.99, 120.00	89.97, 89.98, 119.99
Resolution range (Å)	116.28–1.60 (1.66–1.60)	116.38–1.60 (1.66–1.60)	116.28–1.60 (1.66–1.60)
Diffraction patterns	58030	81966	30491
Total reflections	74240034 (5006769)	85504320 (5774126)	43172014 (2903342)
Unique reflections	32733	32733	32733
Multiplicity	2268 (1572.5)	2612.2 (1813.5)	1318.9 (911.9)
Completeness (%)	100 (100)	100 (100)	100 (100)
Mean *I*/σ(*I*)	17.57 (8.72)	21.94 (12.07)	13.47 (11.03)
Wilson *B* factor (Å^2^)	17.55	15.43	18.07
*R*_split_	0.680 (1.431)	0.677 (1.123)	1.181 (1.061)
CC_1/2_	0.993 (0.974)	0.990 (0.986)	0.981 (0.973)
CC*	0.998 (0.993)	0.998 (0.997)	0.996 (0.993)
Refinement			
Reflections used in refinement	32597 (2491)	32588 (2487)	32591 (2766)
*R*_work_	0.2291 (0.2737)	0.2310 (0.2934)	0.2103 (0.1811)
*R*_free_	0.2537 (0.3125)	0.2555 (0.3584)	0.2464 (0.2259)
Reflections used for *R*_free_	1612 (134)	1616 (134)	1543 (140)
No. of non-H atoms			
Total	2352	2351	2293
Macromolecules	2119	2051	1987
Ligands	5	5	26
Solvent	228	295	280
Average *B* factor (Å^2^)			
Overall	21.37	21.86	19.02
Macromolecules	20.52	20.79	17.87
Ligand	28.83	21.41	20.91
Solvent	29.09	29.29	27.02
R.m.s. deviations			
Bond lengths (Å)	0.005	0.010	0.007
Bond angles (°)	0.869	1.187	0.953

## Data Availability

All coordinate and structure-factor files have been released in the Protein Data Bank as entries 9tkx, 9tky and 9tld.
